# Time‐ and dose‐related pathological changes in knee osteoarthritis rat model induced by monosodium iodoacetate

**DOI:** 10.1002/ame2.70037

**Published:** 2025-06-12

**Authors:** Wei Pu, Qi Liu, Shuyan Xue, Siyuan Li, Nan Nan, Yang Liu, Huiqin Hao

**Affiliations:** ^1^ Third Clinical College Shanxi University of Chinese Medicine Jinzhong China; ^2^ Basic Laboratory of Integrated Traditional Chinese and Western Medicine Shanxi University of Chinese Medicine Jinzhong China; ^3^ Engineering Research Center of Cross Innovation for Chinese Traditional Medicine of Shanxi Province Jinzhong China; ^4^ College of Basic Medical Sciences Shanxi University of Chinese Medicine Jinzhong China

**Keywords:** knee osteoarthritis, micro‐CT, monosodium iodoacetate, pathological change

## Abstract

Knee osteoarthritis (KOA) is a chronic degenerative disease. Monosodium iodoacetate (MIA) induction is the most commonly used therapeutic effect evaluation and mechanism of action research model; we observed a lack of standardization and uniformity in current model building methods, which led us to conduct this study.

**Background:**

The aim was to investigate the time‐ and dose‐related changes in the behavioral and pathological characteristics in the MIA‐induced KOA model rat.

**Methods:**

MIA (40, 50, and 60 mg/mL) was injected into the left joint of male Sprague–Dawley rats. After 2 weeks, the changes in the KOA rat model were observed by behavioral evaluation, imaging‐level evaluation, and histological‐level evaluation. The changes were also compared after 40‐mg/mL MIA injection for 2 and 6 weeks.

**Results:**

MIA‐induced bone surface defects, osteophyte hyperplasia around the articular rim, increased subchondral bone density, thinning of the sparse trabecular bone, structural disorder, and local clustering were observed. The degree of injury gradually increased with the increase in MIA concentration. After 6 weeks, subchondral bone density and sparse trabecular bone increased in the KOA model.

**Conclusions:**

The severity of the model also increased significantly with the changes in dose and time. In dose‐dependent experiments, this study revealed that 40 mg/mL was the optimal dose to induce significant pathological changes without causing undue discomfort or death in animals. This dose may induce pathological changes stably and is suitable for long‐term observation.

## Graphical abstract

Time‐ and dose‐related pathological changes of knee osteoarthritis rat model induced by monosodium iodoacetate.

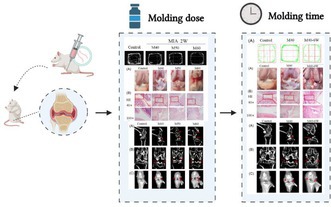

## 1 INTRODUCTION

Knee osteoarthritis (KOA) is a chronic degenerative disease that seriously affects the quality of life and is the leading cause of disability globally as society ages rapidly. In 2010, KOA was the fourth most disabling disease in the world. Studies have shown that the prevalence of KOA is 18% in China, and its prevalence in men and women is 11% and 19%, respectively. The prevalence of KOA in women is significantly higher than that in men.^1^, ^2^ Studies have predicted that by 2050, an estimated 642 million people will have KOA, and the numbers will increase by 74.9% from 2020 to 2050.^3^ The pathogenesis of KOA is very complex, affecting factors such as age, sex, diet, body mass, disease history, genes, living environment, and lifestyle.^4^ The course of the disease is slow and complex. The symptoms in the final stage of the disease are significant pain, stiffness, and limited joint mobility, leading to joint apraxia.

The basic pathological changes in KOA include articular cartilage degeneration and loss, synovial thickening, ligament and joint capsule lesions, and joint margin and subchondral bone hyperplasia.^5^, ^6^ The main clinical manifestations are knee pain, swelling, joint stiffness, limited movement, and even joint deformity. According to the guidelines for diagnosis and treatment,^7^ specific treatment plans should be adopted based on to the patient's individual conditions, symptoms, and signs to improve the quality of life. However, currently this disease is clinically treated mainly using non‐steroidal anti‐inflammatory drugs, but long‐term use will produce gastrointestinal bleeding, perforation, cardiovascular disease, and other adverse reactions.^8^, ^9^

Therefore, new drugs must be produced to improve clinical treatment plans. However, the establishment of KOA animal models is a prerequisite for the study of new medicines.

Monosodium iodoacetate (MIA) is a glycolysis inhibitor that inhibits the activity of glyceraldehyde‐3‐phosphate dehydrogenase in chondrocytes,^10^ leading to energy metabolism disorders in chondrocytes and ultimately to chondrocyte death and cartilage matrix degradation.^11^ MIA can also affect proteoglycan metabolism and inhibit proteoglycan synthesis in patellar and tibial platforms.^12^, ^13^ Progressive degeneration of articular cartilage after MIA injection, including chondrocyte necrosis, proteoglycan loss, and collagen fiber breakage, accompanied by synovial inflammation^14^, ^15^ and subchondral osteosclerosis, is highly similar to human osteoarthritis (OA).

Although the MIA‐induced OA model has been widely used, the MIA dose varies greatly among different studies; the relationship and differences between dose and pathological changes have not been fully determined. At present, systematic studies on pathological changes at different time points in MIA‐induced OA models are limited, especially the lack of in‐depth understanding of the pathological characteristics and evolution process of early OA. There were differences in MIA injection dose, injection site, and animal strain among different studies, which affected the reproducibility and comparability of the model. The instability of the model may affect the efficacy of the drug, and therefore the advantages and disadvantages of different treatment schemes cannot be evaluated. The aim of this study was to systematically investigate the time‐ and dose‐dependent pathological changes in the MIA‐induced OA rat model of the knee joint to bridge the aforementioned research gap. This study aims to develop the standard operating procedure of the KOA model, standardize and unify the KOA model construction method, and eliminate the influence of the model on experimental results.

Therefore, KOA rat models induced by MIA at different concentrations and time points were developed in this experiment, and the pathological changes in articular cartilage were observed and evaluated using behavioral detection, imaging evaluation, and tissue sectioning methods. The aim of this study was to investigate the time‐ and dose‐related changes in behavioral and pathological characteristics in the MIA‐induced KOA rat model, providing reference and assistance for researchers who wish to use the KOA animal models.

## 2 MATERIALS AND METHODS

### 2.1 Materials

*Experimental animals*: 40 specific pathogen‐free male Sprague–Dawley SD rats aged 8–10 weeks, weighing 180–220 g, were purchased at Beijing HFK Bioscience Co., Ltd, and kept in the Laboratory Animal Center of Shanxi University of Traditional Chinese Medicine. The feeding environment was as follows: temperature, 22°C–25°C; relative humidity, 40%–70%; 12‐h light–dark cycle. All rats were able to move, eat, and drink freely. This study was approved by the Experimental Animal Ethics Committee of Shanxi University of Traditional Chinese Medicine (2021DW214).

### 2.2 Methods

#### 2.2.1 Grouping and processing methods

Forty SD rats were randomly divided into a control group (control), a 40‐mg/mL 2W model group (M40), a 50‐mg/mL 2W model group (M50), a 60‐mg/mL 2W model group (M60), and a 40‐mg/mL 6W model group (M40‐6W). MIA powder (C15047184, Shanghai Maclin Biochemical Technology Co., Ltd) was prepared with normal saline into a solution of different concentrations. After isoflurane anesthesia administration, the joints of the rats were skinned and disinfected and fixed on the operating table in a supine position. The left knee joint of the rats was fleeced to expose the knee space, and joint cavity puncture was performed from the most lateral part of the subpatellar patellar tendon using a microsampler; then 50 μL of MIA solution was injected. After injection, the knee joint of the rats was moved to facilitate the absorption of the drug. The left knee joint of the rats in the control group was injected with the same amount of normal saline solution. After 14 days, the behavioral activities, mental state, skin changes in the knee joints and toes, and water and diet status were observed, and behavioral measurements and arthritis index scores (Markin) were performed on the rats.

#### 2.2.2 Behavioral detection

The knee diameter of each group was recorded weekly using an electronic vernier caliper after MIA injection. The knee joint is kept in a straight and upright position, and the inner and outer apex of the femur is the maximum width of the knee joint. The maximum width of the left knee joint was measured using a vernier caliper.

##### Paw mechanical withdrawal threshold

Von Frey fibers (NC12775‐99, YunYan Instrument, China) were used to detect the mechanical withdrawal threshold of the “Up&Down” method, and the 50% Paw Withdrawal Threshold (PWT) of the affected limb was calculated.^16^, ^17^

##### Thermal pain threshold

The positive reaction time of rats was recorded using a hot plate instrument (YLS‐6B, Zhongshi Technology, China), and the average value of three measurements (5‐min interval) was taken as the paw withdrawal latency (PWL).^18^

##### Gait experiment

A DigiGait animal gait detection and analysis system (MSI‐DIG‐RT, Mouse Specifics, USA) was used to force rats to move on the running belt for 20 s, and the footprint contact area was recorded.^19^

##### Open field experiment

A SuperMaze open field analysis system (XR‐Xmaze, XinRuan Technology, China) was used to record the walking path of rats within 5 min to reflect the joint mobility.^20^ After each test, the rats were put back into the cage, and the bottom and side walls of the laboratory were disinfected as the test for the next rat could not be carried out until the laboratory was dry and odor‐free.

#### 2.2.3 Imaging‐level evaluation

The left knee joint of rats was scanned using micro‐CT (model: IRIS PET/CT, French Inviscan). Experimental parameters of rat computed tomography (CT) scanning were voltage, 80 kV; current, 1 mA; projection times, 2000; exposure time, 56 ms; resolution, 60 μm; and a continuous scanning mode. The scan results were quantitatively analyzed for bone microstructure, and the proximal tibial plateau was selected as the region of interest. The measured results bone volume/tissue volume (BV/TV) fraction, trabecular thickness (Tb.Th), and bone surface area/bone volume (BS/BV) ratio were used to reconstruct the scanned images.

#### 2.2.4 Histological evaluation

After the knee joint specimens were fixed in 4% paraformaldehyde solution for 48 h, they were decalcified in 10% ethylenediaminetetraacetic acid decalcified solution for ~4 weeks, dehydrated and embedded in wax. All the samples were sectioned vertically (5 μm thick) in a coronal plane using a paraffin microslicing machine (Leica, CM1850). Hematoxylin–eosin (HE) staining and Safranin O‐Fast Green staining were performed. The cartilage structure, cells, matrix, and tide line of knee slides were observed under an optical microscope. The sections were observed, and their images were obtained using a microscope. Using the principle of the double‐blind experiment, three researchers who were blinded to the grouping rated the results of the slices, using the average of the scores as the final result. The histological changes in KOA were evaluated using the Markin scoring method.^21^ The higher the final score, the more serious the injury to the articular cartilage was.

#### 2.2.5 Enzyme‐linked immunosorbent assay

According to the manufacturer's instructions, the levels of matrix metalloproteinase (MMP3) (RA20503, Bioswamp, China) and cartilage oligomeric matrix protein (COMP) (RA20863, Bioswamp) in the serum of rats were detected using the enzyme‐linked immunosorbent assay (ELISA) method.

#### 2.2.6 Statistical analysis

SPSS software (version 20.0, USA) from IBM Corporation was used for data analysis, and the results were expressed as mean ± standard deviation. A one‐way analysis of variance (ANOVA) was used to compare differences between groups (least significant difference *t*‐test for data with homogeneity of variance and Dunnett's T3 test for data with heterogeneity of variance). *p* < 0.05 was considered statistically significant. GraphPad Prism software, version 8.0.2, was used for constructing graphs.

## 3 RESULTS FOR DIFFERENT MIA CONCENTRATIONS

### 3.1 The behavior in KOA rats induced by different MIA concentrations

**FIGURE 1:**
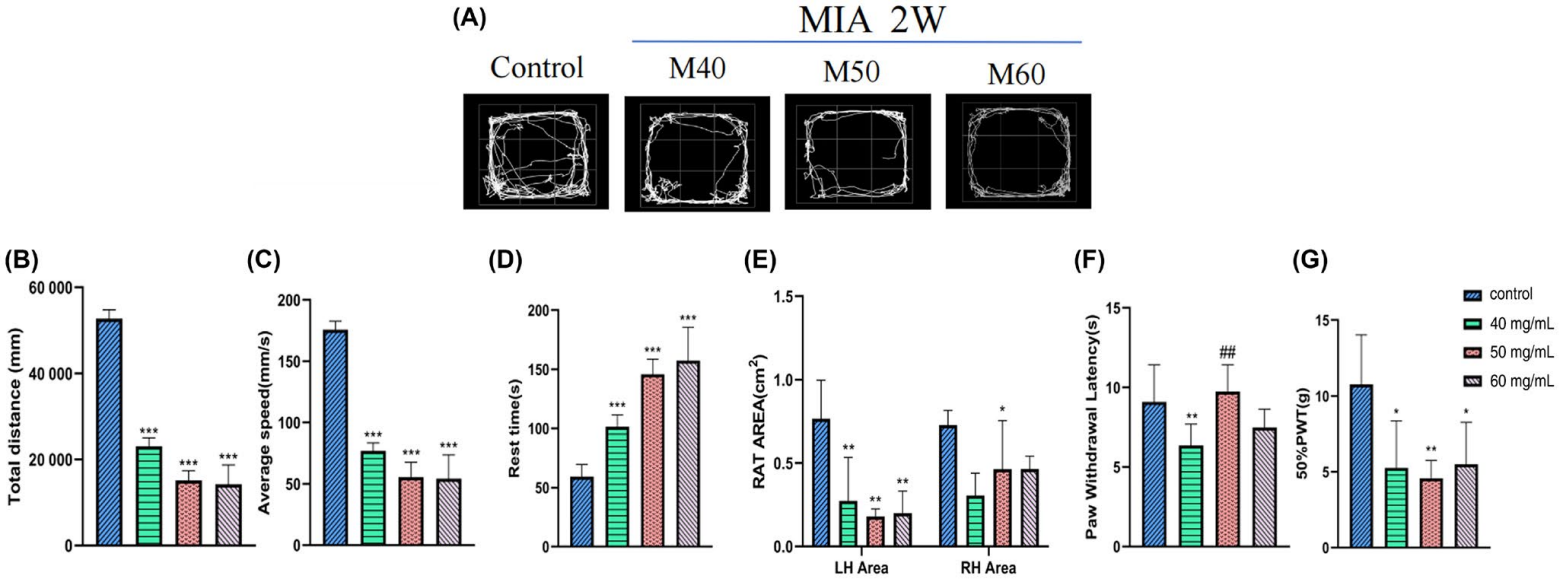
The behavior in KOA (knee osteoarthritis) rats induced by different concentrations of MIA (monosodium iodoacetate). (A) Pathway maps of KOA rats in the open field experiment. (B) The total distance in the open field experiment. (C) The average motor speed in the open field experiment. (D) Resting time in the open field experiment. (E) The bilateral footprint contact area in the gait experiment. (F) The paw withdrawal latency. (G) The 50% paw withdrawal threshold. Compared to control, *p < 0.05, **p < 0.01, and ***p < 0.001. Compared to M40, ^##^ *p* < 0.01.

In the open field experiment (Figure 1A–D), compared with the control group, the total distance and average speed of autonomous movement in both M40 and M50 groups decreased and exhibited a certain dose‐dependent trend (*p* < 0.001). The results of the gait experiment (Figure 1E) showed that the left footprint contact area of the model group was significantly different from that of the control group, with statistical significance. In the heat pain threshold experiment (Figure 1F), the PWL of the M40 group was significantly reduced compared with the control group (*p* < 0.01). In the plantar mechanical pain threshold experiment (Figure 1G), 50% PWT in the model group decreased compared with the control group (*p* < 0.05), showing a statistical difference.

### 3.2 Pathological changes in KOA rats induced by different MIA concentrations

**FIGURE 2:**
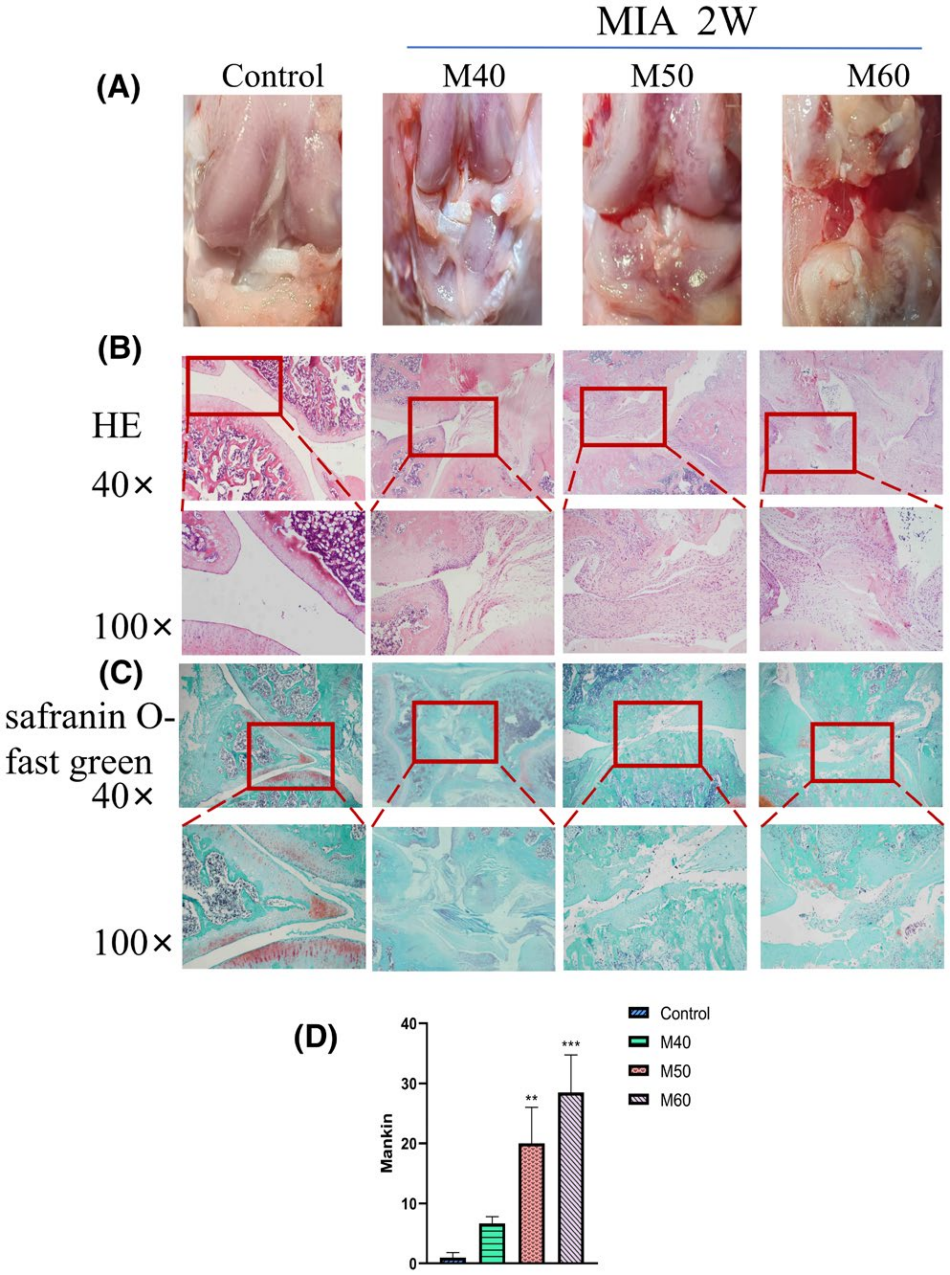
Pathological changes in KOA (knee osteoarthritis) rats induced by different concentrations of MIA (monosodium iodoacetate). (A) Stereoscopic representation of KOA rats induced by MIA at different concentrations. (B) Representative images of HE (hematoxylin–eosin) staining in KOA rats induced by MIA at different concentrations. (C) Representative images of Safranin O‐Fast Green staining in KOA rats induced by MIA at different concentrations. (D) Markin scores of KOA rat cartilage sections induced by MIA at different concentrations. Compared to control, **p < 0.01 and ***p < 0.001.

Compared with the control group, the results of stereomicroscopic observation (see Figure 2A) showed that the articular surface of the M40 group was rough; the articular surface of the M50 concentration model group was rough and ulcerated; and the articular surface of the M60 group revealed joint effusion, the tibial plateau was rough and ulcerated, and the surrounding tissues were degenerated.

Staining and scoring of tissue sections (Figure 2B,C): HE and Safranin O‐Fast Green staining showed that the articular cartilage surface was flat and chondrocytes were arranged neatly in the control group. The staining of cells and cartilage matrix was normal, and there was no decrease. In the MIA group, the cartilage surface was rough and fused, became thin and discolored, and revealed fibrosis; the chondrocytes were irregular and clustered; the deep chondrocytes exhibited hypertrophy and vacuolar changes; and some cartilage staining decreased. The degree of cartilage damage increased with the increase in MIA concentration. Figure 2D shows that the Markin score of the cartilage tissue section indicated that compared with the control group, scores of the M40, M50, and M60 groups were all increased and exhibited a certain dose‐dependent trend (*p* < 0.01, *p* < 0.001).

### 3.3 The CT results of knee joints in KOA rats induced by different MIA concentrations

**FIGURE 3:**
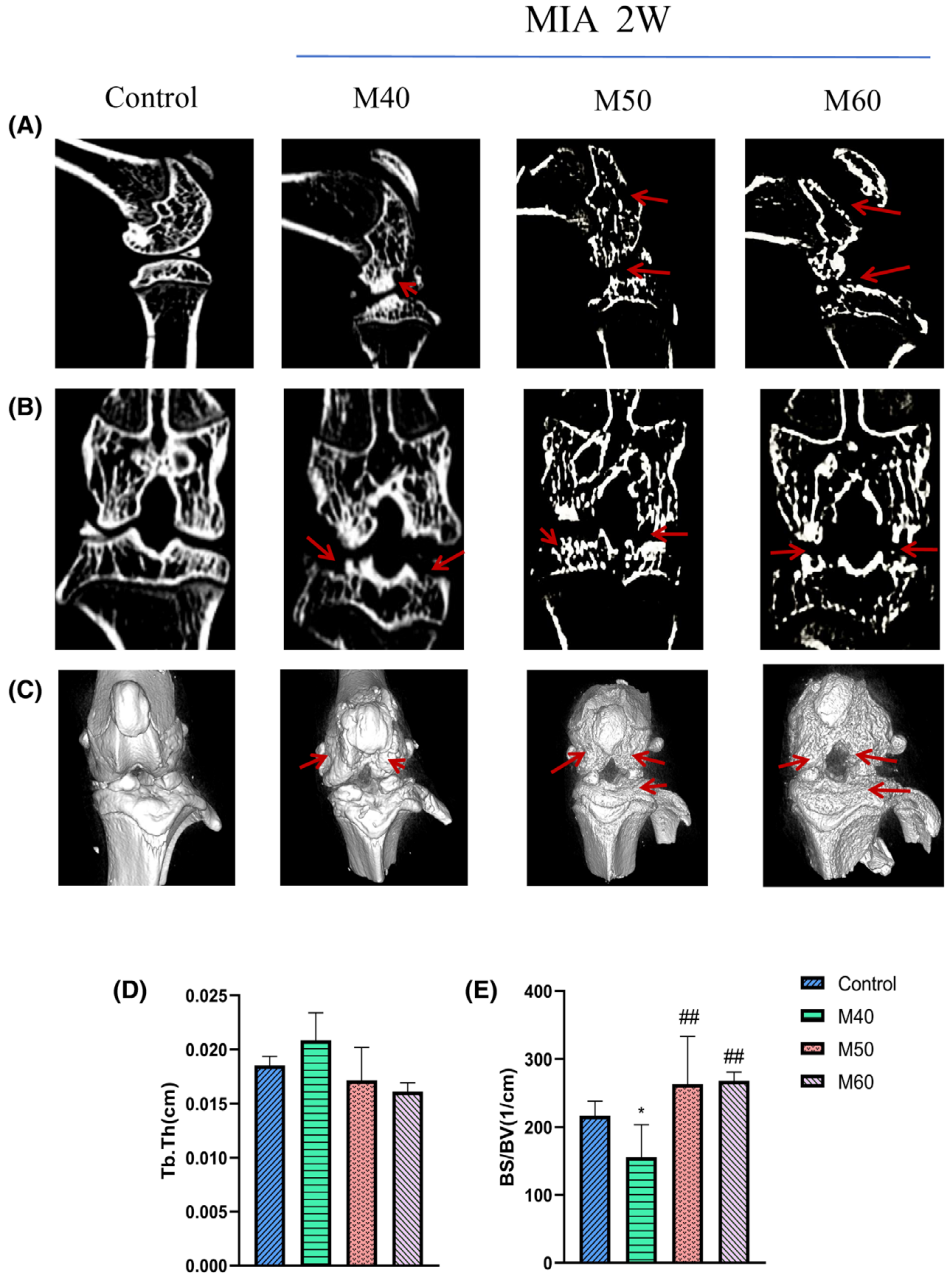
The CT (computed tomography) results of knee joints in KOA (knee osteoarthritis) rats induced by different concentrations of MIA (monosodium iodoacetate). (A) Representative sagittal CT images of knee joints of KOA rats induced by MIA at different concentrations. (B) Representative coronal CT images of the knee joint of rats with different concentrations of MIA. (C) Representative 3D (three‐dimensional) stereoscopic images of rat knee joints with different concentrations of MIA. (D) Trabecular thickness of knee joint in KOA rats induced by MIA at different concentrations (Tb.Th). (E) Knee bone surface area to bone volume ratio in KOA rats induced by MIA at different concentrations (BS/BV). Compared to control, *p < 0.05; compared to M40, ^##^ *p*<0.01.

At the micro‐CT imaging level (Figure 3A–C), the changes in the CT sagittal plane, coronal plane, and three‐dimensional (3D) stereoscopic plane of KOA rat knee joint induced by MIA at different concentrations were observed. Compared with the control group, the femoral surface and tibial plateau of the M40, M50, and M60 groups were partially eroded, and the area of the M60 group was maximum. Compared with the control group (Figure 3D,E), the BS/BV value of M40 was significantly decreased (*p* < 0.05). Compared with the M40 group, the BS/BV value of the M50 and M60 groups was significantly increased (*p* < 0.01).

### 3.4 Expression of serum inflammatory factors in KOA rats induced by different MIA concentrations

**FIGURE 4:**
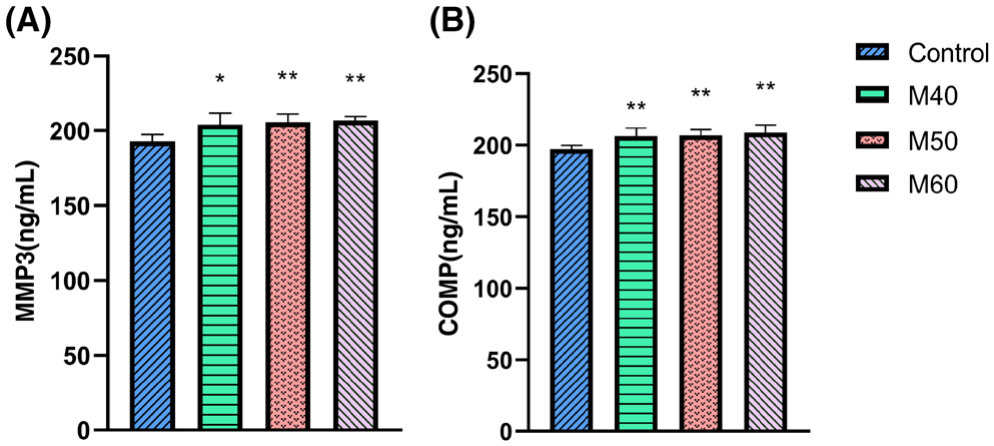
Expression of serum inflammatory factors in KOA (knee osteoarthritis) rats induced by different concentrations of MIA (monosodium iodoacetate). (A) Different concentrations of MIA induce the expression of MMP3 (matrix metalloproteinase) inflammatory factors in the serum of KOA rats. (B) Different concentrations of MIA induce the expression of COMP (cartilage oligomeric matrix protein) inflammatory factors in the serum of KOA rats. Compared to control, *p < 0.05 and **p < 0.01.

Compared with the control group (Figure 4A,B), the levels of MMP3 and COMP (cartilage oligomeric matrix protein) in the M40, M50, and M60 groups were significantly increased.

## 4 RESULTS FOR DIFFERENT TIMES AFTER MIA INJECTION

### 4.1 Behavioral experimental results of MIA‐induced KOA rats over time

**FIGURE 5:**
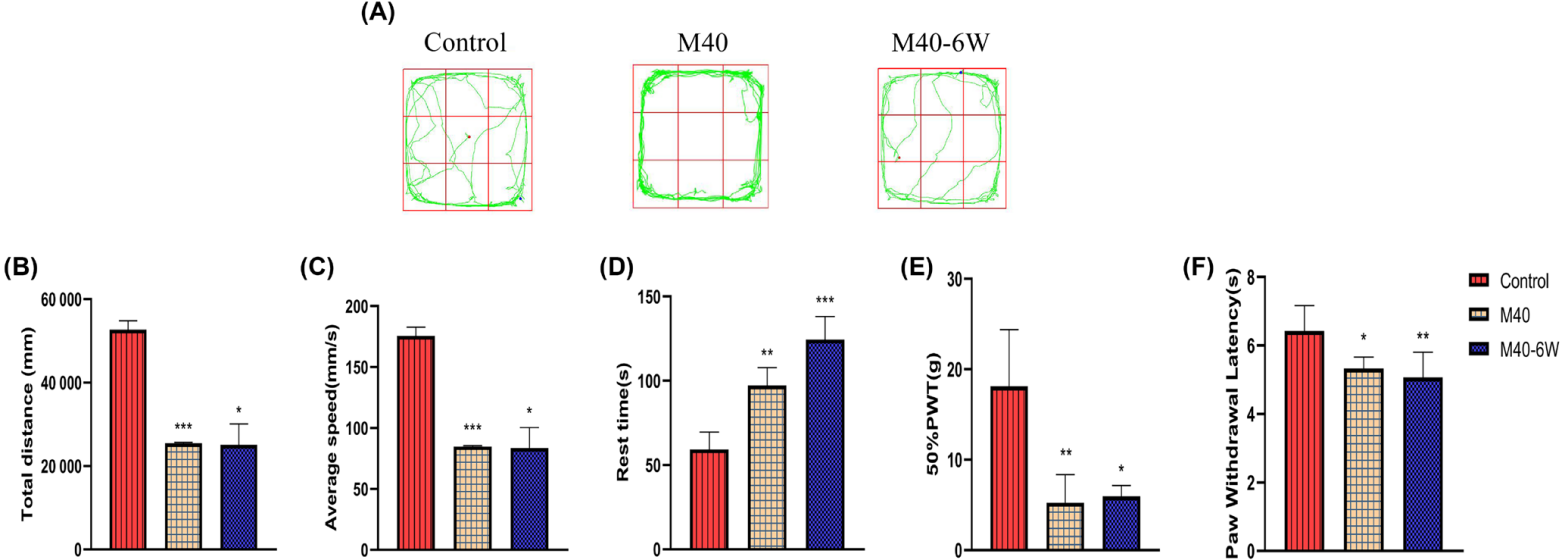
Behavioral experimental results of MIA (monosodium iodoacetate)–induced KOA (knee osteoarthritis) rats over time. (A) Path map of MIA‐induced KOA rats in the open field experiment over time. (B) The total movement distance of MIA‐induced KOA rats over time in the open field experiment. (C) The mean motion velocity of MIA‐induced KOA rats in the open field experiment with time. (D) The resting time of MIA‐induced KOA rats in the open field experiment with time. (E) The 50% paw withdrawal threshold in MIA‐induced KOA rats over time. (F) Time‐varying paw withdrawal latency in MIA‐induced KOA rats. Compared to control, *p<0.05, **p<0.01, and ***p< 0.001.

In the open field experiment (Figure 5A–D), compared with the control group, the total distance of autonomous movement in the M40 and M40‐6W groups decreased, the average speed decreased, and the rest time increased.

In the heat pain threshold experiment (Figure 5F), PWL in the M40 and M40‐6W group was significantly reduced compared with the control group (*p* < 0.01). In the plantar mechanical pain threshold experiment (Figure 5E), 50% PWT in the model group decreased compared with the control group (*p* < 0.05), showing a statistical difference.

### 4.2 Pathological changes in MIA‐induced KOA rats over time

**FIGURE 6:**
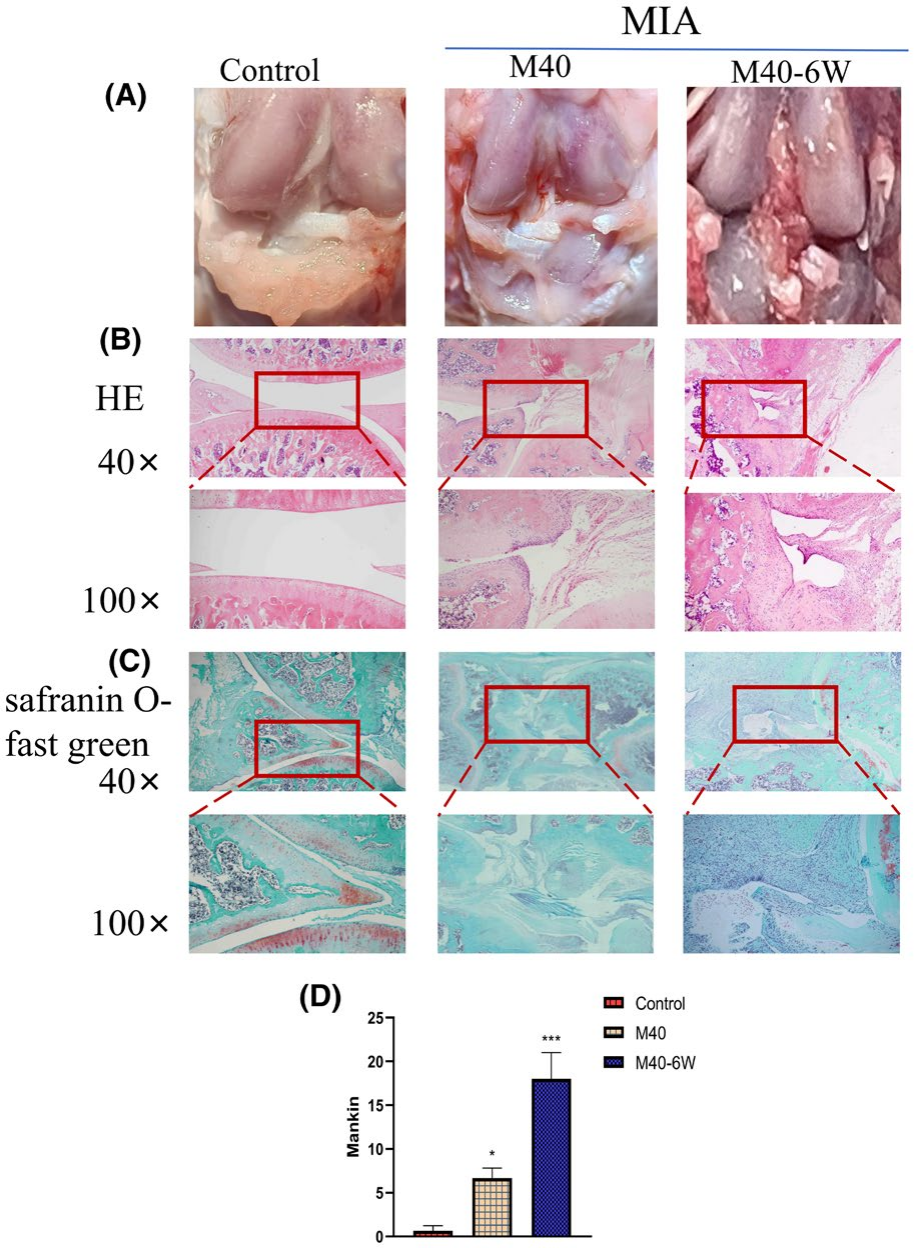
Pathological changes in MIA (monosodium iodoacetate)–induced KOA (knee osteoarthritis) rats over time. (A) Stereographic representation of MIA‐induced KOA rats over time. (B) Representative images of MIA‐induced KOA rats with HE (hematoxylin–eosin) staining changes over time. (C) Representative images of MIA‐induced KOA rats with Safranin O‐Fast Green staining changes over time. (D) Markin scores of MIA‐induced cartilage sections in KOA rats changed over time. Compared to control, *p<0.05 and ***p<0.001.

Stereomicroscopic observation (Figure 6A) showed that, compared with the control group, the articular surface of the M40 group was rough, and the articular surface of the M40‐6W group was rough, with joint effusion and surrounding tissue degeneration.

Stained sections were observed under the microscope (Figure 6B,C), and the articular surfaces in the control group were smooth and the chondrocytes were arranged neatly. The staining of cells and cartilage matrix was normal, and there was no decrease. The cartilage surface in the MIA model group was rough, cracked, and thinner than that in the control group. The morphology of the chondrocytes was irregular and clustered, and the cartilage surface was fibrotic. With the extension of modeling time, the degree of cartilage damage became more serious. Markin score of cartilage tissue slice showed that compared with the control group (Figure 6D), the scores of the M40 and M40‐6W groups all increased, and exhibited a certain time‐dependent trend (*p* < 0.001).

### 4.3 CT results of knee joints in MIA‐induced KOA rats over time

**FIGURE 7:**
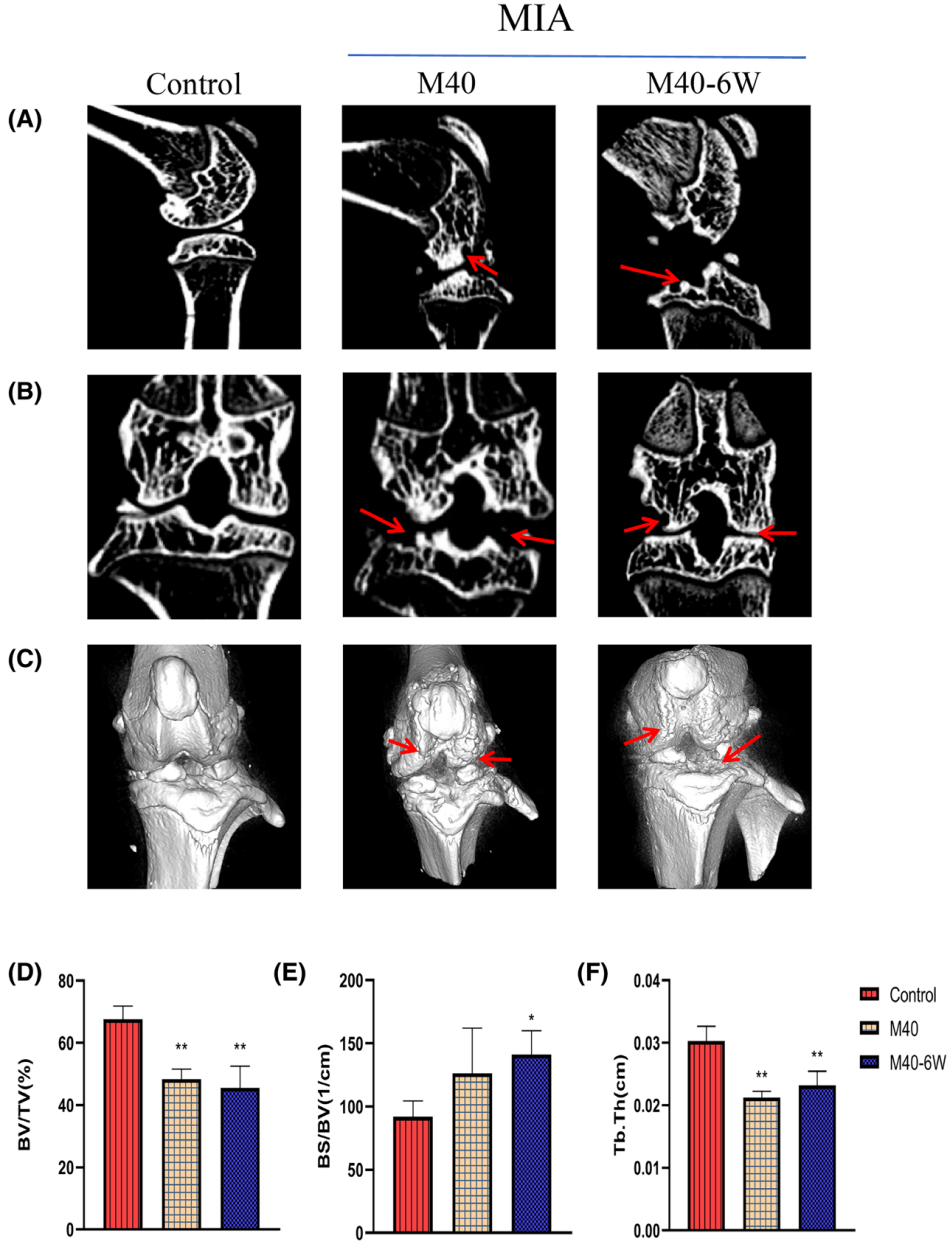
The CT (computed tomography) results of knee joints in MIA (monosodium iodoacetate)–induced KOA (knee osteoarthritis) rats over time. (A) Representative CT sagittal images of the knee joint of MIA‐induced KOA rats over time. (B) Representative coronal CT images of the knee joint of MIA‐induced KOA rats over time. (C) 3D (three‐dimensional) stereoscopic representative images of the knee joint of MIA‐induced KOA rats over time. (D) Knee bone volume/tissue volume (BV/TV) fraction of MIA‐induced KOA rats over time. (E) Ratio of knee bone surface area to BV over time (BS/BV) in MIA‐induced KOA rats. (F) Changes in knee trabecular thickness (Tb.Th) over time in MIA‐induced KOA rats. Compared to control, *p <0.05 and **p<0.01.

Micro‐CT imaging level of small animals (Figure 7A–F) shows the CT sagittal, coronal, and 3D changes in the knee joint of MIA rats over time. Compared with the control group, there was partial erosion of the femoral surface in the M40 group, and the erosion area of the tibial plateau and femoral surface in the M40‐6W group increased; bone surface defects, osteophyte hyperplasia at the joint periphery, subchondral bone density increase, and bone trabecular thinning were observed. Compared with the control group, BS/BV in the M40 and M40‐6W groups significantly increased (*p* < 0.05). BV/TV and Tb.Th in the M40 and M40‐6W groups significantly increased (*p* < 0.01).

### 4.4 Expression of serum inflammatory factors in MIA‐induced KOA rats over time

**FIGURE 8:**
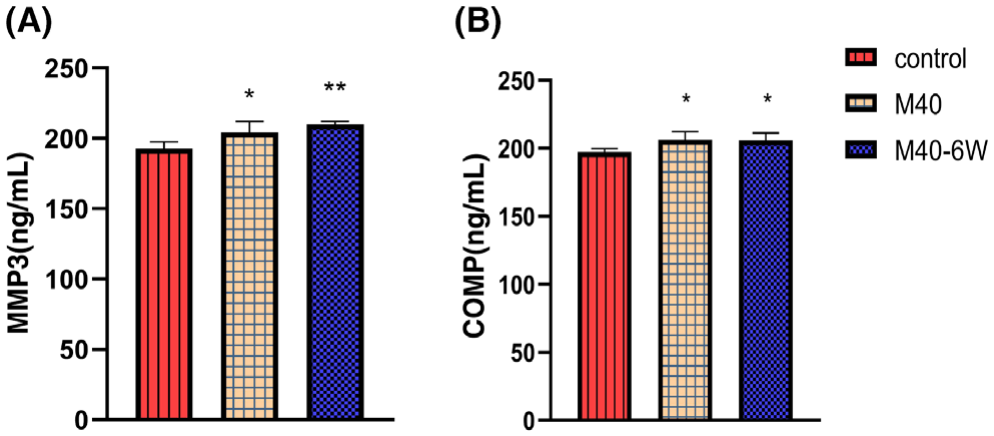
Expression of serum inflammatory factors in MIA (monosodium iodoacetate)–induced KOA (knee osteoarthritis) rats over time. (A) The expression of MMP3 (matrix metalloproteinase) inflammatory factors in MIA‐induced KOA rats over time. (B) The expression of COMP (cartilage oligomeric matrix protein) inflammatory factors in MIA‐induced KOA rats over time. Compared to control, *p < 0.05 and **p < 0.01.

ELISA results showed that compared with the control group (Figure 8A,B), the expression levels of MMP3 and COMP in the M40and M40‐6W groups increased.

## 5 DISCUSSION

KOA is a chronic degenerative disease affecting the knee, and it is characterized by cartilage degradation, muscle atrophy, local inflammation of the joint, and osteophyte formation. It can cause joint stiffness, pain, deformation, and loss of function in patients, seriously reducing their quality of life and being one of the main causes of disability.^22^, ^23^ KOA can lead to a loss of balance in the local stable environment of the joint, producing relevant pro‐inflammatory factors. These inflammatory factors not only activate related signaling pathways but also directly destroy the structural composition of cartilage, extracellular matrix, synovium, and so on, forming a vicious cycle and accelerating the progression of the disease.^24^ At present, KOA animal models can be divided into spontaneous and induced types.^25^ The pathogenesis of the spontaneous KOA model is very similar to that of clinical models. The disadvantage is that the modeling cycle is long and the economic cost is high. Inducible KOA models include surgical and drug‐induced models. The advantages are direct induction through sterile surgery or drug injection, high reproducibility, and a short modeling cycle.^26^

Articular cartilage is a highly specialized and poorly regenerated tissue composed of an extracellular matrix, which is synthesized only by sparsely distributed resident chondrocytes. Articular cartilage injury usually leads to OA, which has three related characteristics: loss of synovial space, cartilage angiogenesis, and osteophyte formation.^27^ Articular cartilage is a tough and flexible load‐bearing connective tissue with unique biological and biomechanical properties. It covers the articular surface of the long bone in the synovial joint. The repair and regeneration ability of articular cartilage is poor. Cartilage is avascular.^28^ Due to the fact that circulation is a crucial part of the normal healing process, a lack of blood supply to the cartilage may inhibit normal responses related to healing. Therefore, the survival of chondrocytes is crucial for maintaining an appropriate cartilage matrix, and damage to chondrocyte function and survival can lead to joint cartilage failure.

During the development of KOA, MMP3 exacerbates joint destruction by degrading proteoglycan, collagen, and extracellular matrix,^29^ and the cartilage matrix gradually degrades due to overexpression of multiple stroma‐destroying enzymes. Excessive release of MMP3 can induce chondrocytes to lose their normal phenotype. Studies have shown that MMP3 is highly sensitive and specific. It is one of the potential biomarkers for diagnosing and examining the progression of KOA.^30^ COMP is an extracellular matrix glycoprotein that is crucial for collagen assembly and extracellular matrix stability. The increased expression of COMP in chondrocytes near the damaged cartilage may be related to self‐repair of the injury or matrix supplementation.^31^ Studies have found that high levels of serum COMP are significantly correlated with early OA, suggesting that serum COMP is likely to be a predictor of OA.^32^

MIA inhibits an inhibitor of glyceraldehyde‐3‐phosphate dehydrogenase in the Krebs cycle. Injection of MIA into the joint cavity can induce loss of proteoglycan matrix and cell death,^33^ causing changes in cartilage matrix, degradation and loss of articular cartilage, synovitis, and other local lesions, leading to joint instability and the development of OA, similar to the pathological changes in human OA. Studies have shown that MIA sensitizes joint pain receptors, making it a useful model for studying the severity of OA and the mechanism of joint pain.^34^

In this study, articular puncture injection was used, and a volume of 50 μL of MIA solution was injected into one knee joint, with concentrations of 40, 50, and 60 mg/mL, respectively. In the second week of induction, rats in the M40, M50, and M60 groups all exhibited reduced mechanical and thermal pain, and movement (Figure 1). The surface of the cartilage was rough and cracks appeared, the surface of the cartilage became thinner, and the morphology of chondrocytes was irregular (Figure 2). Both the tibial plateau and femoral cartilage exhibited erosion (Figure 3). In the sixth week of induction, the erosion area of the tibial plateau and the femoral surface in the M40‐6W group increased, and the bone surface was defective (Figure 7), which indicated that the pathological injury degree of knee joint increased with induction time. In summary, after MIA induction, the surface damage of the joint cartilage in the model group was significant, with structural disorder and a certain dose and time dependence. This study designed a rat KOA model induced by MIA at different concentrations and time points. By measuring the changes in the intrinsic structural characteristics of the subchondral bone and conducting histological evaluation, we found that intra‐articular injection of MIA caused cartilage matrix degradation, such as surface defects and shallow or no staining of cartilage. MIA also induces inflammation, improves COMP and MMP3 levels, and reduces the expression of cartilage‐specific proteins with prolonged modeling time. At the same time, the imaging results (Figures 3 and 7) show that MIA accelerates the remodeling of the subchondral bone, characterized by a decrease in subchondral bone density and the disorder in cartilage structure and morphology. Although our results are limited to animal models, the comprehensive pathological features of MIA‐induced KOA described in this paper can provide preclinical evidence of the treatment methods of clinical KOA and basic scientific guidance for the study and at the same time contribute to the study of the overall pathogenesis of the disease and provide a reference for the clinical diagnosis of the staging and severity of knee joint diseases. Further research is needed on the pathological mechanisms underlying the occurrence and development of KOA.

Although this study provided time‐ and dose‐related pathological changes for a rat model of KOA induced by MIA, there are still some limitations. Rats and humans differ in anatomy, physiological function, and disease course, and the findings may not be fully applicable to humans. The study induced only unilateral knee OA and could not reflect the systemic effect of bilateral OA. The MIA‐induced OA model is an acute chemical injury that differs from the chronic degenerative process of human OA. Different MIA doses may lead to different pathological changes, which need to be further verified. The small sample size of this study may affect the statistical validity of the results, and the observation time was short, which could not fully reflect the long‐term progress of OA. Finally, this study did not deeply explore the molecular mechanism of MIA‐induced KOA and lacked therapeutic intervention. In conclusion, this study provides valuable data for the MIA‐induced rat model of KOA but needs to be combined with other models and approaches to more fully understand the pathological mechanisms of KOA and develop effective treatment strategies.

## 6 CONCLUSION

In this study, our results suggest that the model with a concentration of 40 mg/mL exhibited milder symptoms at 2 weeks, whereas the models with concentrations of 50 and 60 mg/mL exhibited more severe symptoms. The progression of the disease worsened at 40 mg/mL until 6 weeks, which is consistent with the human disease course and helpful for studying the overall disease development process. Therefore, it is recommended to choose the MIA model with a concentration of 40 mg/mL for modeling.

## AUTHOR CONTRIBUTIONS

**Wei Pu:** Conceptualization; data curation; formal analysis; investigation; methodology; validation; writing – original draft. **Qi Liu:** Data curation. **Shuyan Xue:** Data curation. **Siyuan Li:** Data curation. **Nan Nan:** Data curation; funding acquisition; supervision. **Yang Liu:** Supervision. **Huiqin Hao:** Funding acquisition; resources; supervision.

## FUNDING INFORMATION

This work was supported by grants provided by the Construction Project of High‐Level Traditional Chinese Medicine Key Discipline of National Administration of Traditional Chinese Medicine (zyyzdxk‐2023022), Key Team of Scientific and Technological Innovation Talents of Shanxi Province with Integrated Traditional Chinese and Western Medicine for Preventing and Treating Rheumatological Diseases (grant number: 202204051002033), Traditional Chinese Medicine + Stem Cell Innovation Project (2024KJZY006), 2023 Shanxi Graduate Research Practice Project (2023KY676), 2023 Graduate Innovation and Entrepreneurship Project of Shanxi University of Traditional Chinese Medicine (2023CX023 and 2023CX027), Science and Technology Innovation Project for University in Shanxi Province (2022L358), and Key Laboratory of Rheumatological and Immunological Diseases Treated by Integrated Chinese and Western Medicine (zyyyjs2024021).

## CONFLICT OF INTEREST STATEMENT

We declare that we have no financial or personal relationships with others or organizations that can inappropriately influence our work. We have read and approved the manuscript. We declare that they have no conflicts of interest.

## ETHICAL APPROVAL

This study was approved by the Experimental Animal Ethics Committee of Shanxi University of Chinese Medicine (Approval ID 2021DW214). The methods were carried out in accordance with the approved guidelines. The study conforms to the ARRIVE guidelines and should be carried out in accordance with the U.K. Animals (Scientific Procedures) Act, 1986 and associated guidelines, EU Directive 2010/63/EU for animal experiments, or the National Institutes of Health guide for the care and use of Laboratory animals (NIH Publications No. 8023, revised 1978).

## Data Availability

Data will be made available on request.
